# Sera from Phylogenetically Related Alligators, Crocodiles and Domestic Chickens Exhibit Comparable Anti-Cancer Activity

**DOI:** 10.3390/cells15090749

**Published:** 2026-04-22

**Authors:** Ofer Binah, Gil Shalev, Gila Maor, Irina Reiter, Inbal Ziv, Aaron Ciechanover

**Affiliations:** 1 Department of Physiology, Biophysics and Systems Biology, Rappaport Faculty of Medicine, Technion-Israel Institute of Technology, Haifa 3109601, Israel; 2 The Rappaport-Technion Integrated Cancer Center (R-TICC) and The Rappaport Faculty of Medicine and Research Institute, Technion-Israel Institute of Technology, Haifa 3109601, Israel

**Keywords:** anti-cancer activity, alligator, crocodile and chicken sera, C5 protein of the Complement system, membrane attack complex (MAC), IgM

## Abstract

Background: Crocodilians rarely develop cancer despite long lifespans and continuous exposure to environmental carcinogens, suggesting robust natural anti-tumor defense mechanisms. Methods: We investigated the anti-cancer activity of sera derived from the phylogenetically related species—alligators, crocodiles, and chickens, and studied their underlying immune mechanisms. The anti-tumor activity of alligator serum was tested in murine models of melanoma and lymphoma. Results: Alligator serum (AS) and its (NH_4_)_2_SO_4_-precipitated fraction (ASa) showed rapid and potent cytotoxicity toward multiple murine and human cancer cell lines while sparing non-malignant human cells. Importantly, ASa attenuated melanoma and lymphoma tumor growth in mice. Electrophysiological analyses in PN71 cancer cells treated with ASa revealed rapid membrane depolarization and formation of high-conductance pores consistent with Complement-mediated membrane attack complex (MAC) activity. Proteomic analyses identified the Complement component C5 as a major protein enriched in active fractions, implicating the Complement system in cancer cell killing. Based on phylogenetic similarity of C5, crocodile and chicken sera exhibit alligator-like comparable anti-cancer activity. Mechanistic studies in chicken serum showed that the anti-cancer activity depends on Ca^2+^ and Mg^2+^ ions, terminal Complement components (C5–C8), and IgM antibodies that initiate Complement activation. Immunodepletion of IgM from CSa significantly reduced cytotoxicity, whereas purified chicken IgM activated human Complement to induce cancer cell death. Conclusions: These findings identify a conserved IgM–Complement immune mechanism capable of selectively targeting malignant cells. The evolutionary conservation and cross-species functionality of this pathway highlight its potential as a bio-inspired strategy for developing novel Complement-based cancer immunotherapies.

## 1. Introduction

A well-known observation is that crocodiles and alligators have been suggested to exhibit a relatively low incidence of cancer based on limited observational reports, offering an opportunity to investigate resistance mechanisms for novel therapies. However, it is important to note that systematic epidemiological data in these species are lacking, and therefore, this notion should be interpreted with caution. Specifically, it remains unclear how crocodiles can live for up to a century without developing malignancies despite constant exposure to harmful factors such as pathogens, decaying food, heavy metals, and radiation, all known carcinogenic factors in humans. Despite these exposures, several reports have proposed that crocodilians may possess robust innate defense mechanisms that contribute to their resilience; however, these observations remain largely descriptive and hypothesis-generating rather than evidence from controlled studies [1,2,3].

Biologically derived molecules have shed light on natural tumor-suppressive mechanisms and have provided a foundation for anti-cancer drug discovery, either as direct therapeutics, as scaffolds for semisynthetic optimization, or as leads that illuminate new mechanistic pathways. A complementary and increasingly active area is the exploration of serum-derived proteins and peptides as sources of cytotoxic or immunomodulatory activities relevant to cancer biology. Indeed, growing evidence supports the central role of biologically derived molecules in shaping modern oncology, both as therapeutic leads and as conceptual frameworks for understanding tumor vulnerability [4]. In addition, serum contains complex mixtures of proteins that participate in host defense, including Complement components, pattern recognition molecules, antimicrobial/host defense peptides, protease inhibitors, and carrier proteins [5,6]. The same biochemical features that enable these molecules to recognize and damage microbial membranes, opsonize targets, or trigger inflammatory cascades may also confer activity against malignant cells.

Crocodilians are of particular interest as sources of potent serum defense activities. Early work demonstrated broad antibacterial activity in American alligator (*Alligator mississippiensis*) serum compared with human serum and showed that heat treatment under conditions commonly used to inactivate Complement activity abolished the antimicrobial effect, supporting an important role for active serum Complement and/or Complement-associated proteins [2,7,8,9]. More recently, reports testing crocodile sera and organ lysates have described measurable cytotoxicity and growth inhibition using multiple cancer cell lines, with evidence that proteinaceous factors contribute to this activity (e.g., reduced effect following heat treatment or protease digestion in some systems). For example, Siddiqui and colleagues [2] reported that serum and selected organ lysates from *Crocodylus palustris* triggered substantial prostate cancer cell death in vitro. Similarly, Jeyamogan et al. [10] described the anti-tumor activities of selected pure compounds identified from the serum of *Crocodylus porosus*, *Malayopython reticulatus*, *Varanus salvator* and *Cuora kamaroma amboinensis*. In addition, crocodile blood extracts were reported to induce apoptosis in A549 lung cancer cells, providing further rationale that crocodilian-blood-derived factors can affect tumor cell survival pathways. Collectively, these and other studies support the broader concept that crocodilian sera contain bioactive molecules with potential relevance to anti-cancer modalities discovery, while also emphasizing the importance of systematic evaluation across species, experimental conditions and non-malignant cells’ controls.

Building on prior observations of crocodilian serum bioactivity and the known abundance of innate and adaptive immune effectors in avian serum, we tested the hypothesis that sera from these phylogenetically close species contain distinct protein/peptide factors capable of reducing cancer cell viability. Specifically, our work aims to decipher whether anti-cancer effects caused by proteinaceous innate immune components, such as Complement activators, provide a foundation for downstream fractionation and identification of the bioactive anti-cancer molecules.

## 2. Materials and Methods

### 2.1. Preparation of Alligator, Crocodile and Chicken Sera and 45% (NH_4_)_2_SO_4_ Fractionation

Crocodile and alligator blood samples were drawn from animals residing in farms in Israel (Crocoloco and Hamat Gader) for routine health monitoring, under the veterinary supervision of the animal farms. Chicken blood was collected immediately upon slaughter (25 mL/animal) for commercial purposes at the Milouot Corporation for the Development of Haifa Bay Settlements Ltd. (https://www.milouot.co.il/). Blood samples were stored on ice for 2 h, clots were then punctured, and samples were further spun 2–3 times at 1000 g for 20 min at 4 °C. Supernatants were collected as a clear serum source, aliquoted to 20 mL stocks and stored at −80 °C. A fully saturated (NH_4_)_2_SO_4_ solution kept at 4 °C, featuring the maximal amount of dissolved (NH_4_)_2_SO_4_ (100%) was used to provide a constant dilution of the (NH_4_)_2_SO_4_ salt used in successive processing of different serum sources. Accordingly, the appropriate volume of this saturated (NH_4_)_2_SO_4_ solution was added to the final dilution of 45% salt. After 30 min on ice, the mixture was spun for 15 min at 13,000 rpm and 4 °C. The sediment was subsequently resuspended in 20 mM HEPES + 150 mM NaCl and fractionated again in 45% (NH_4_)_2_SO_4_. The final sediment was collected in half of the original volume of HEPES/NaCl buffer. The precipitate (denoted as **ASa**) fractions were dialyzed against 1000 volumes of the same HEPES/NaCl buffer for 3 × 1 h in 6.4 cm dialysis membrane tubing (MWCO 12–14 K, Spectrum Medical Industries Inc., Laguna Hills, CA, USA), having a cutoff of 12,000–14,000 Daltons. Finally, ASa fractions were tested for their anti-cancer activity.

### 2.2. Recording Membrane Potential and Membrane Current from PN71 Cancer Cells

Membrane potential and membrane current were recorded from PN71 cancer cells placed over Matrigel-coated glass coverslips. Following plating, a recovery period of at least two days was allowed before performing electrophysiological experiments. The experiments were conducted in external Tyrode’s solution containing (in mM) 140 NaCl, 5.4 KCl, 1 MgCl_2_, 2 sodium pyruvate, 1 CaCl_2_, 10 HEPES, and 10 glucose (pH 7.4 adjusted with NaOH). Axopatch 200B, Digidata 1322, and pClamp10 (Molecular Devices, Sunnyvale, CA, USA) were used for data amplification, acquisition and analysis. Signals were digitized at 10 kHz and filtered at 2 kHz. Patch pipettes with resistances of 4–7 MΩ were pulled from borosilicate glass capillaries (Harvard Apparatus, Holliston, MA, USA). Analysis was performed using MATLAB 2016b software (MathWorks, Natick, MA, USA). The patch pipette solution contained (mM): 120 KCl, 1 MgCl_2_, 3 Mg-ATP, 10 HEPES, and 10 EGTA titrated to pH 7.2 with KOH and adjusted to 290 mOsm with saccharose.

### 2.3. Generation of iPSC-CMs

In the current study, we used the previously described human-induced Pluripotent-Stem-Cell-derived Cardiomyocyte (iPSC-CM) clone FSE [11]. iPSC differentiation into cardiomyocytes was performed according to a previously described directed differentiation by modulating Wnt/β-catenin signaling protocol [12,13]. iPSCs were cultured on Matrigel (GFR, BD Biosciences, Franklin Lakes, NJ, USA)-coated 6-well plates in mTeSR1 medium (Stemcell Technologies, Vancouver, BC, Canada) for 5–6 days. To initiate differentiation, cells were incubated with 1 mL/well Versene solution (Invitrogen, Life Technologies, Woburn, MA, USA) at 37 °C for 5 min and seeded on Matrigel-coated 12-well plates in mTeSR1 medium. The medium was replaced daily, and after 2 days, when the monolayer of cells reached 100% confluence, the medium was changed to RPMI supplemented with B27 minus insulin (Invitrogen, Life Technologies, Woburn, MA, USA) containing 10 µM CHIR99021; this day was counted as day 1 of differentiation. On the next day (day 2 of differentiation), the medium was changed to RPMI supplemented with B27 minus insulin. On day 4, the medium was changed to RPMI supplemented with B27 minus insulin, containing 10 µM of IWP-4. On day 6, the medium was changed to RPMI supplemented with B27 minus insulin. Finally, from day 8 onwards, the medium was supplemented with RPMI with B27 complete supplement (Invitrogen, Life Technologies, Woburn, MA, USA). A total of no less than ten different differentiation protocols were utilized throughout this study. To enrich cardiomyocyte fraction in the culture, a MACS magnetic labeling-based cell sorting kit was used (130-110-188, Miltenyi Biotec, Bergisch Gladbach, Germany). To generate beating monolayers, 25–50 K enriched cardiomyocytes were seeded in a ~3 mm diameter circle on glass coverslips, formed by a 40 µL Matrigel droplet.

### 2.4. Monitoring Cell Viability

#### 2.4.1. MTT Assay

Cancer and normal cells were plated in a 96-well plate (30,000 cells/well) in triplicate and placed in an incubator to adhere. ASa was applied at 1 mg/mL for 18 h, and subsequently, the MTT colorimetric assay determining cell viability was performed by administration of 25 µL of a ready-to-use solution of the MTT reagent (Sigma, St. Louis, MI, USA; M2128) for 2 h at 37 °C. Next, 100 µL/well of dissolving buffer was administered (20 g of SDS dissolved in 100 mL 50% DMF/DDW, and adjusted to pH = 4.7) for an additional 2 h of incubation at 37 °C. The developed color was measured by an ELISA plate reader at 570 nm. The obtained values were processed by blank subtraction, dividing the OD value of each treatment by that of the untreated cells, and finally multiplying by 100 to express cell viability in % change.

#### 2.4.2. [H^3^]-Thymidine Incorporation

To determine the effects of the anti-cancer fractions on the proliferation activity, cells were incubated for the last 3 h in the presence of 1 mCi/mL [methyl-H^3^]-thymidine. Cells were washed with PBS and methanol to increase membrane permeability. Soluble thymidine was washed out by rinsing with 1% TCA (tri-chloro-acetic-acid) 3 × 5 min. Cells were then washed with DDW and dissolved with 0.3 M NaOH for 15 min. The level of cell proliferation was expressed in relation to the level of the Control FBS-treated cells.

### 2.5. Purification/Enrichment of Anti-Cancer Fractions (Methods for Table 2)

Enrichment for serum proteins responsible for the anti-cancer effect was carried out by running independent chromatographic steps using an ÄKTA™ Avant chromatography system (28976337, Cytiva, Malborough, MA, USA) equipped with a Superdex 200 Increase Size Exclusion Chromatography (SEC) 3.2/300 column (28990946, Cytiva), and a RESOURCE™ S Cation Exchange Chromatography (CEX) column (17118001, Cytiva) run either independently or as a stepwise purification process starting with the CEX step followed by the SEC step. Accordingly, the CEX step was processed with Buffer A (HEPES 50 mM pH = 7.4) and Buffer B (HEPES 50 mM pH = 7.4 supplemented with 3 M NaCl). The SEC step was processed with an eluant solution consisting of HEPES 50 mM pH = 7.4 supplemented with 150 mM NaCl. All purification steps were carried out with either pre-fractionated sera ((NH_4_)_2_SO_4_-precipitated) or outcomes of the CEX step once the stepwise purification scheme was implemented. All the resulting protein fractions were tested individually for their anti-cancer effect.

### 2.6. Assessing the Abundance of C5 Complement System Protein by Mass Spectrometry (MS)

Neighboring fractions (eluted successively) featuring anti-cancer effect (measured by the MTT protocol) were pooled and further subjected to MS analyses at The Smoler Proteomics Center, the Technion-Israel Institute of Technology, Haifa, Israel. All masses obtained were crossed with crocodilian proteins enlisted in the UniProt protein database. Table 1, summarizing the interpretation of all peptides revealed, points to 15 common peptides that appeared in all independent fractionations executed, of which 14 peptides were attributed to crocodile proteins, whereas 12 peptides were annotated to the Complement C5 protein (A0A1U8DWU5 at UniProt).

### 2.7. Generation of Customized Polyclonal Antibodies Raised Against Chicken C5-C8 Proteins (Methods for Figure 5)

A series of epitopes attributed to chicken Complement proteins was selected by the “Kyte & Doolittle” and the “Hopp & Woods” algorithms, available at https://web.expasy.org/protscale/. These epitopes were used for raising a series of polyclonal antibodies applied to the immuno-depletion studies. Accordingly, peptides were synthesized and further conjugated to KLH at Sigma-Aldrich, Rehovot, Israel. Subsequently, the resulting KLH-conjugated peptides were used to immunize rabbits (2 rabbits/conjugate), and final bleedings were subjected to affinity purifications. Affinity-purified antibodies were employed for the immuno-depletion protocol. The epitopes selected for the chicken Complement C5–C8 proteins are listed in Table 1.

## 3. Results

### 3.1. The Anti-Cancer Effect of Alligator Serum

#### 3.1.1. Alligator Serum Kills Mouse and Human Cancer Cell Lines

**Figure 1:**
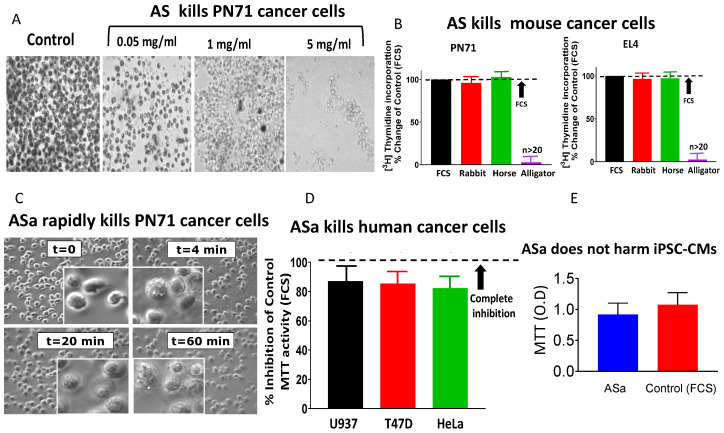
Alligator serum induces rapid and selective cytotoxicity in cancer cells. (**A**) Dose-dependent cytotoxic effect of alligator serum (AS) on PN71 mouse myeloma cells following 2 h incubation. Cell viability was assessed by the MTT assay; dark staining indicates metabolically active cells. (**B**) AS (2 mg/mL, 2 h) selectively kills mouse cancer cell lines PN71 (myeloma) and EL4 (lymphoma), whereas fetal calf serum (FCS, n > 20), rabbit serum (n = 3), and horse serum (n = 3) show no cytotoxic effect. The anti-cancer effect was measured by monitoring cell proliferation determined by [^3^H]-Thymidine incorporation into DNA. [^3^H]-Thymidine incorporation in the presence of FCS was defined as 100%. (**C**) Time-dependent killing of PN71 cells by (NH_4_)_2_SO_4_-precipitated alligator serum (ASa, 1 mg/mL). Representative images show progressive morphological deterioration, with complete loss of cell integrity within 60 min. (**D**) ASa induces cytotoxicity in human cancer cell lines (U937, n = 5; T47D, n = 6; HeLa, n = 5), as measured by the MTT assay after 18 h incubation. (**E**) ASa does not affect the viability of non-malignant human iPSC-derived cardiomyocytes (iPSC-CMs) under identical conditions. In (**A**,**C**) magnification is 400×.

The fundamental finding of this study is that *Alligator mississippiensis* serum (AS) kills a broad range of mouse cancer cells. Figure 1A shows the dose-dependent anti-cancer effect measured by the MTT assay (see Materials and Methods for details) for PN71 mouse myeloma cancer cells following 2 h incubation with AS. Increasing AS protein concentration from 0.05 to 5.0 mg/mL reduced cell viability (e.g., fewer live cells stained in black), resulting in complete cell death. This specificity of AS is demonstrated by the observation that, whereas it kills the murine cancer cell lines PN71 and EL4 (a murine T lymphoblast originated from a lymphoma induced in a C57BL mouse by 9,10-dimethyl-1,2-benzanthracene), fetal calf, rabbit, and horse sera are ineffective (Figure 1B). In these experiments, the anti-cancer effect is determined by the ability of AS to inhibit DNA synthesis, as determined by [^3^H]-Thymidine incorporation into DNA. To partially purify the active anti-cancer component in AS, we performed a calibration protocol aiming at determining the minimal (NH_4_)_2_SO_4_ concentration retaining the anti-cancer activity upon precipitation. As we found that 45% (NH_4_)_2_SO_4_-precipitated serum (ASa) and crude serum (AS) present identical anti-cancer effects, all subsequent experiments were performed using ASa, which rapidly kills PN71 cancer cells (Figure 1C). ASa also kills the human cancer cell lines (Figure 1D) U937 (histiocytic lymphoma), T47D (breast carcinoma) and HeLa (cervical carcinoma), but does not harm human-induced Pluripotent-Stem-Cell-derived Cardiomyocytes (iPSC-CMs) (Figure 1E). The larger sample size for the alligator serum (AS) group reflects the central focus of the study, which was to rigorously characterize its anti-cancer activity across multiple independent preparations and experiments. In contrast, rabbit and horse sera were included as negative biological controls to demonstrate lack of AS cytotoxicity.

As the first step in testing the potential translational anti-cancer applications of ASa, in preliminary experiments we tested the ability of ASa to attenuate melanoma and lymphoma in murine models. Notably, these exploratory experiments were intended as proof-of-concept (POC) preliminary studies, rather than definitive efficacy studies. Murine melanoma was induced by intracutaneous injections of murine B16 (melanoma-derived) cells into the back skin of C57/bl mice; see details in the Supplementary Materials and Methods and Figure S1. Briefly, the morphological appearance of the melanoma shows large, damaged areas in the ASa-treated melanoma, compared to the well-preserved FCS-injected tumor (Figure S1A). As shown in Figure S1B, on day 8 of ASa treatment, it decreased the melanoma tumor volume by ~50% compared to the control and FCS-injected tumors. Next, murine syngeneic peritoneal lymphoma model was generated by injecting 0.5 × 10^6^ EL4 cells intraperitoneally (i.p.) into inbred strain C57Bl/6 mice; see details in Materials and Methods in the Supplementary Materials and Methods and Figure S2. As shown in Figure S2, 4 days after ASa injection, the EL4 cell count decreased by ~80% compared to the cell count in the FCS-injected lymphoma mouse.

#### 3.1.2. The Involvement of the Complement System in the Anti-Cancer Effect of ASa

**Figure 2:**
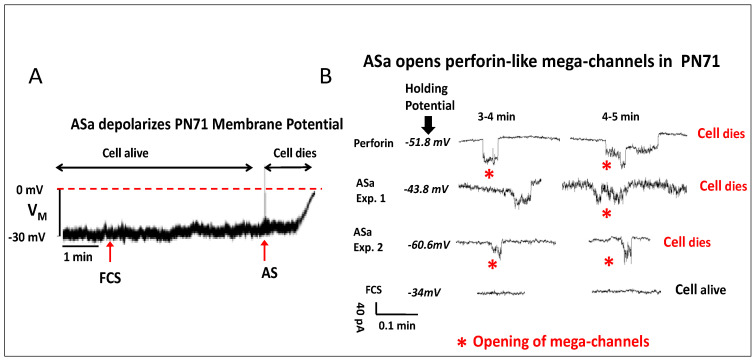
Alligator serum causes Complement-dependent membrane depolarization and pore formation in PN71 cancer cells. (**A**) Membrane potential (V_M_) recording show rapid depolarization toward 0 mV following ASa treatment, but not FCS application, consistent with loss of membrane integrity and cell death. (**B**) Voltage clamp recordings illustrate the formation of high-conductance membrane pores in ASa- but not in FCS-treated cells, consistent with membrane attack complex (MAC) activity. The PN71 were maintained (“holding potential”) at their respective membrane potential measured using the current clamp mode.

Previous studies have shown that the membrane attack complex (MAC) of the Complement system causes formation of ion-conducting channels of high conductance in the order of pico-Siemens (pS) [14,15,16]. To test whether the Complement system is involved in ASa-dependent anti-cancer activity, we recorded from ASa-treated PN71 cancer cells the membrane potential (V_M_) and membrane current (I_M_) using the patch clamp technique. As illustrated in Figure 2A, whereas application of fetal calf serum (FCS) to the recording bath did not affect V_M_, a highly diluted ASa caused, within a few minutes, V_M_ depolarization towards 0 mV, culminating in cell death. Strong evidence for Complement involvement (i.e., MAC) in ASa anti-cancer effects is the induction of perforin-like mega-channels in PN71 cancer cells (Figure 2B). Opening these high-conductance channels rapidly enables large ion influx/efflux, causing cell demise. In support of the notion that the Complement system is involved in the anti-cancer effect of alligator serum, the C5 protein, an important component of the Complement system, was found: (i) in all serum-derived protein fractions resolved via different chromatographic separations (e.g., gel filtration, ion exchange chromatography, hydrophobic interaction; Table 2) enriching for the anti-cancer effect; (ii) C5 was the most abundant Complement component protein identified by MS (more than 100 different peptides collectively covering the entire protein length); and (iii) incubating ASa at 55 °C treatment for 20 min abolished anti-cancer activity.

**Table 2:** Complement C5 is involved in the anti-cancer activity of crocodilian sera. Pre-fractionation of crocodilian sera by (NH_4_)_2_SO_4_ precipitation protocol was followed by different biochemical purification steps, collectively aimed at enriching for the proteins governing anti-cancer activity. All the resulting protein fractions were tested for their anti-cancer effect against PN71 cells (MTT assay). The neighboring fractions (eluted successively) featuring anti-cancer effects were first pooled and further subjected to mass spec analyses. Eventually, 15 common masses were retrieved in all the purification outcomes exhibiting the anti-cancer effect (red): 14 masses out of those were attributed to specific crocodile proteins, whereas 12 peptides were annotated to the Complement C5 protein (A0A1U8DWU5 at UniProt).

Serum Injected	Biochemical Approaches Aimed at Enriching for the Cell Killing Activity	No. of Peptides Detected in Each Pooled Protein Fractions Featuring the Cell Killing Activity	Common Peptides Detected in All the Protein Fractions	The Protein Identity of the Common Peptides Revealed
***Alligator mississippiensis***	Affinity Chromatography step executed with a column packed with an immobilized antibody blocking the cell killing activity	1103		
	A Size-Exclusion Chromatography (Superdex 200 column)	2370	236	
***Crocodylus niloticus***	A Cation Exchange column followed by a Size-Exclusion step (Superdex 200)	1389	38	
	A Size-Exclusion Chromatography (Superdex 200)	2546	**15**	12 peptides were annotated to the Complement C5 protein

### 3.2. Sera of Crocodiles and Chickens Also Display Anti-Cancer Effect

**Figure 3:**
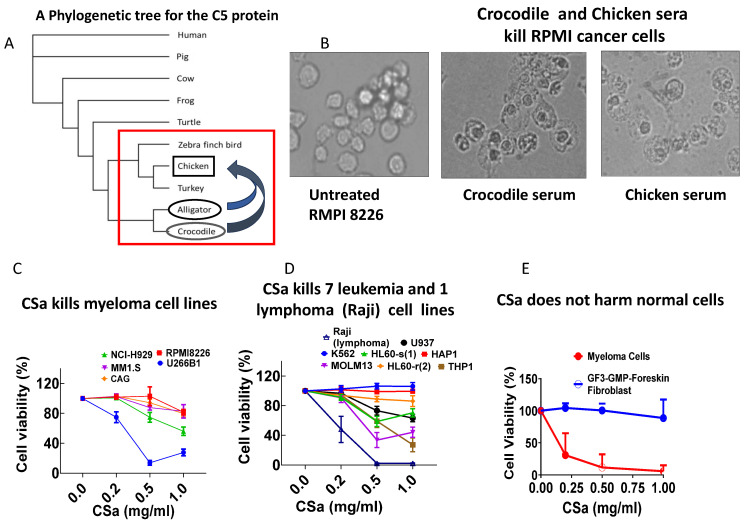
Anti-cancer activity is conserved in crocodile and chicken sera. (**A**) Phylogenetic analysis of the Complement component C5 demonstrates close evolutionary relatedness among alligators, crocodiles, and chickens, marked by the red box. (**B**) Sera from crocodiles and chickens induce rapid cytotoxicity in human RPMI 8226 myeloma cells following 2 h incubation. Magnification 400×. (**C**) Dose-dependent cytotoxicity of chicken serum fraction (CSa) across multiple myeloma cell lines, with variable sensitivity. Experiments were performed on fully adhered cells at 80% confluency, incubated at different CSa concentrations overnight at 37 °C. Following the incubation period, cell viability was measured by the MTT protocol. U266B, n = 10; RPMI 8226, n = 3; NCI-929, n = 6; MM1.S, n = 5; CAG, n = 8. U266B1 (blue symbols) features the highest sensitivity to CSa with a significant difference from the other tested myeloma cell lines, calculated by two-way ANOVA, *p* < 0.0001. (**D**) CSa kills (measured by the MTT protocol) 7 different leukemia cell lines and 1 lymphoma cell line (Raji). K562, n = 5; HAP1, n = 3; HL60-s (1), n = 8; MOLM13, n = 5; 3; HL60-r (2), n = 6; U937, n = 7; THP1, n = 5; Raji, n = 4. HL-60 cells were received from 2 different sources and were therefore labeled HL60-s (1) and HL60-r (2). The Raji cell line shows the highest sensitivity to CSa, with a significant difference from the other tested cell lines. (**E**) CSa kills the RPMI8226 (n = 5) myeloma cancer cell line, but does not affect non-cancerous human GF3-GMP-Foreskin Fibroblasts (kindly provided by Accellta Ltd., Haifa, Israel) (n = 5).

Based on our findings in alligator serum, we tested the hypothesis that phylogenetically related species also display similar anti-cancer activity. To accomplish this goal, we performed a phylogenetic analysis aimed at identifying species with the closest C5 protein sequence to that of alligators (Figure 3A). Indeed, the first support for this hypothesis emerges from our findings showing that serum from crocodiles, whose C5 sequence is closely related to that of alligators, exhibits alligator-like anti-cancer activity and kills RPMI 8226 cancer cells within 2 h (Figure 3B). Because alligators and crocodiles are protected species, based on the prominent relatedness of the alligator, crocodile and chicken (*Gallus gallus*) C5 sequence (Figure 3A), we tested whether the chicken serum (CS) exhibits alligator-like anti-cancer activity. Indeed, in support of the C5 hypothesis, CS kills RPMI 8226 cancer cells within 2 h (Figure 3B). Next, we found that the 45% (NH_4_)_2_SO_4_-precipitated fraction of chicken serum (**CSa**) also kills five myeloma cell lines, exhibiting different sensitivities to CSa, where U266B1 (blue symbols) is the most sensitive (Figure 3C). In addition, the seven leukemic cell lines tested also display different sensitivities to CSa, with the lymphoma cell line Raji being the most sensitive (Figure 3D). In contrast, CSa does not affect the viability of human dermal fibroblasts (HDFs), but does cause dose-dependent killing of the human myeloma cell line RPMI8226 (Figure 3E).

### 3.3. The Involvement of the Complement System in Chicken Serum Anti-Cancer Activity

**Figure 4:**
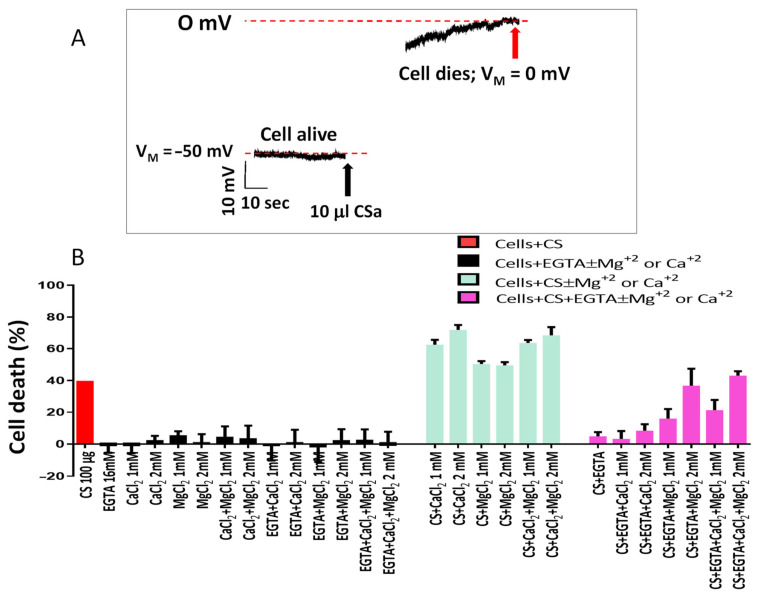
Chicken serum cytotoxicity depends on Complement activation and Mg^2+^ and Ca^2+^ ions. (**A**) The effect of CSa on the membrane potential (V_M_) of a single PN71 cell. At the beginning of the experiment, the membrane potential of the cell was −49 mV. Addition of 10 μL CSa depolarized V_M_ until reaching 0 mV, indicating cell death (~5 min after recording began). (**B**) The dependence of the anti-cancer effect of CSa on Mg^2+^ and Ca^2+^ ions supports the role of the Complement system as a co-factor indispensable for the anti-cancer effect. A set of experiments (n = 3) demonstrates that chelation of Mg^2+^ and Ca^2+^ ions by EGTA reduced the anti-cancer activity of CSa by 88 ± 3% compared to non-EGTA-treated cells. Further addition of Mg^2+^ completely restored the anti-cancer activity. The bars present percentage of cell death under the different treatments. Cell death was normalized to the same treatment carried out in cells treated with heated serum. The columns and bars represent mean ± SEM. Note that the *Y*-axis is “Cell death”.

**Figure 5:**
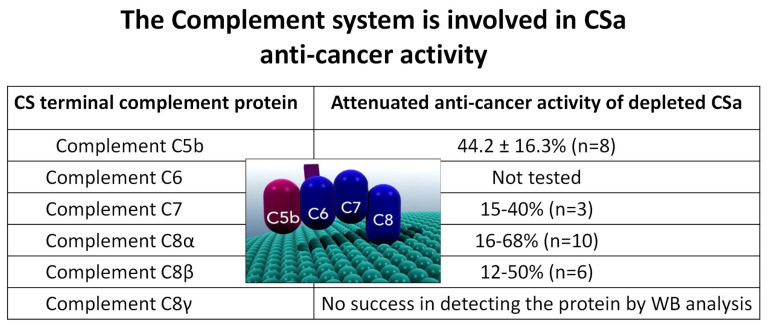
Terminal Complement components are required for CSa-mediated cytotoxicity. Immuno-depletion of Complement proteins (C5–C8) using specific polyclonal antibodies significantly reduces the anti-cancer activity of CSa. These findings demonstrate that Complement proteins act cooperatively and are individually required for effective cytotoxicity.

As shown for the alligator serum (ASa, Figure 2A), CSa also causes a rapid (~40 s) depolarization of the membrane potential (V_M_) toward 0 mV, culminating in cell death, thus supporting the notion that the Complement system contributes to the anti-cancer activity of CSa (Figure 4A). To further support Complement involvement, we tested the dependency of CSa killing on the presence of Mg^2+^ and Ca^2+^ ions which are essential for proper activity of any Complement pathway/arm (classical, alternative and lectin). As shown in Figure 4B, compared to the CaCl_2_- and MgCl_2_-containing culture medium (green bars), the anti-cancer activity was blocked in the presence of 16 mM EGTA, and partially restored by increasing MgCl_2_ and CaCl_2_ concentrations. To evaluate the requirement of each Complement protein for CSa anti-cancer effects, we used a series of affinity-purified polyclonal antibodies raised against selected epitopes of MAC-related proteins (C5–C8; see Materials and Methods for further details) for immune depletion assays (IDs) of specific Complement proteins. As illustrated in Figure 5, CSa anti-cancer activity was attenuated to various extents upon depleting different Complement proteins, providing additional proof for Complement-mediated cancer cell death. Regardless of the contribution of any tested Complement protein to the anti-cancer effect, these experiments show that Complement proteins work in concert, while being individually indispensable for executing the cell death program.

### 3.4. IgM Antibodies Participate in the Anti-Cancer Effect of CSa

**Figure 6:**
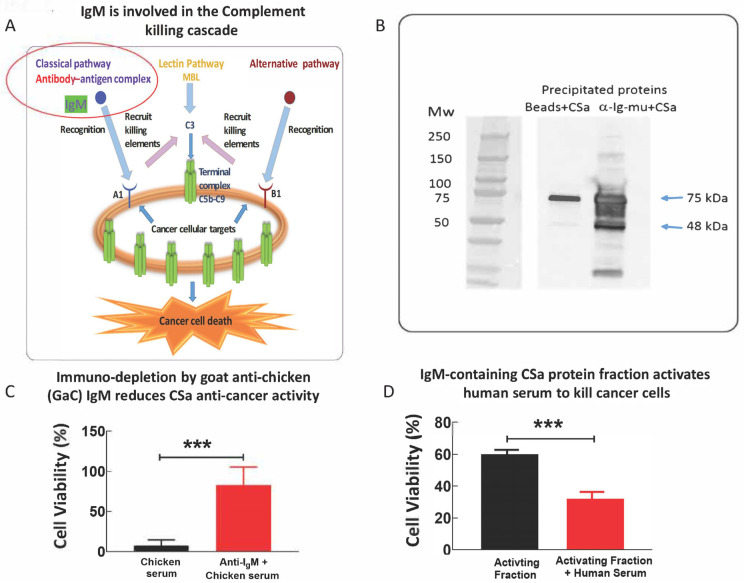
IgM antibodies initiate Complement-mediated cancer cell killing. (**A**) Schematic model illustrating IgM-mediated activation of the classical Complement pathway leading to MAC formation. (**B**) Western blot confirming depletion of chicken IgM using the anti-chicken biotin Ig-mu antibody. (**C**) Immuno-depletion of chicken IgM by commercial goat anti-chicken (GaC) IgM attenuated anti-cancer activity by 82 ± 24%. Six identical immunoprecipitation experiments show significant attenuation of the anti-cancer activity of CSa by a GaC-biotin IgM Ab (SAB3700240, Sigma-Aldrich) vs. CSa treated by streptavidin-coated agarose beads alone, when applied to RPMI 8226 cells at 10, 25 and 50 μg/30,000 cells. Unpaired t-test was performed on the values of cell viability, n = 6, *** *p* < 0.001. Columns and bars represent mean ± SEM. (**D**) CSa-derived IgM-enriched fraction activates human serum (HS) to kill cancer cells. The IgM activating fraction was applied to human U266B1 myeloma cells (25 μL/50,000 cells) in the absence (black bar) or presence (red bar) of 40% human serum (*v*/*v*). The cells were incubated for 16 h at 37 °C, and cell viability was then determined using a CellTiter-Glo assay (G7570, Promega, Madison, WI, USA). Unpaired t-test was performed on the values of cell viability, n = 6, *** *p* < 0.001 (two-tailed *t*-test).

IgM initiates the classical Complement pathway by recruiting the C1q protein via binding to a certain antigen (Figure 6A). Accordingly, to determine whether the anti-cancer effect of CSa is mediated by IgM molecules, we tested whether immuno-depleting chicken serum IgM by using a commercial anti-chicken Ig-mu antibody (targeting the constant heavy chain of IgM molecules; see details in the Materials and Methods), affects CSa-dependent anti-cancer activity. As illustrated in Figure 6B, the commercial antibody precipitates chicken IgM, as expected. Accordingly, immuno-depleting chicken IgM inhibits CSa anti-cancer activity by 82 ± 24% (Figure 6C), thereby supporting the role of IgM as a mediator of CSa anti-cancer activity, being a potent initiator of the classical pathway.

To decipher whether purified chicken IgM antibodies can trigger human-serum-mediated anti-cancer activity, chicken serum was fractionated using a 10 mL IgM purification column (prepacked with Sepharose high-performance resin), and relevant protein fractions were pooled and further fractionated by a Size Exclusion Chromatography step. Accordingly, IgM-containing fraction (10 µg) was applied to human U266 myeloma cells (30 µL/50,000 U266 cells) in the absence or presence of 40% human serum (HS, *v*/*v*) for 3 h at 37 °C. As shown in Figure 6D, the presence of human serum and IgM-containing fraction collectively activated the human Complement system, which resulted in strong anti-cancer activity.

## 4. Discussion

The present study demonstrates that alligator serum and its 45% (NH4)_2_SO_4_ fraction (ASa) exhibit potent and selective anti-cancer activity against various murine and human cancer cell lines. Importantly, this killing effect is mediated by the Complement system, as evidenced by both electrophysiological and biochemical analyses. Moreover, the discovery that chicken serum (CS) and its corresponding fraction (CSa) share these anti-cancer properties situates this phenomenon within a broader evolutionary continuum of innate immune defense among reptiles and avians. Collectively, these findings uncover a conserved Complement-mediated mechanism with potential translational applications in anti-cancer immunotherapy. Our findings illustrate that both crude serum (AS) and its 45% (NH_4_)_2_SO_4_-precipitated fraction (ASa) rapidly kill cancer cells (Figure 1A). Across multiple assays, AS/ASa rapidly kills murine cancer lines (PN71 myeloma; EL4 lymphoma) and diverse human cancer lines (e.g., U937, T47D, HeLa), while sparing a non-cancerous human cell type (iPSC-derived cardiomyocyte; Figure 1E) under the tested conditions. Collectively, these findings support the presence of serum factor(s) with preferential cytotoxicity toward malignant cells. Notably, this rapid cytotoxic effect was observed exclusively with crude AS, but not with rabbit, horse or fetal calf sera (Figure 1B).

### 4.1. In Vivo Anti-Cancer Activity of ASa Supports Potential Translational Outcome/Promise

Injections of ASa to mice bearing B16 melanoma tumors cause pronounced intra-tumoral necrosis (Figure S1A), resulting in a substantial reduction in tumor volume, ~50% compared with FCS-treated controls (Figure S1B). Further, i.p. ASa injection to EL4-cell-induced peritoneal lymphoma caused an 80% decrease in EL4 cell count compared to the cell count in FCS-injected lymphoma mouse (Figure S2). These in vivo anti-tumor effects strengthen the physiological relevance of the in vitro cytotoxicity and argue that Complement-driven cytolysis can operate when the active serum fraction is delivered locally.

### 4.2. Experimental Evidence Showing That the Complement MAC Underlies ASa Killing Mechanism

Several independent observations converge on a Complement-dependent anti-cancer cytolytic mechanism. First, patch clamp recordings show ASa-induced depolarization of the membrane potential toward ~0 mV within minutes after application, culminating in cell death. Second, ASa triggers very large conductance perforin-like mega-channels in the order of pS consistent with pore formation [16], whereas fetal calf serum does not. These electrophysiological signatures are compatible with membrane perforation by the Complement MAC, which assembles from C5b–C9 and forms pores [17,18,19,20,21]. These large pores disrupt ionic homeostasis, causing rapid depolarization, lysis and extensive leakage of intracellular components, ultimately resulting in cell death (Figure 2A,B). Third, the elimination of the cytotoxic effect by heat treatment (55 °C, 20 min) and the fact that this effect requires the presence of the Mg^2+^ and Ca^2+^ ions, are consistent with heat-labile Complement components being obligatory for cytotoxicity. While this study does not directly quantify Complement regulators’ expression, the observed selectivity (cancer cells killed, whereas iPSC-CMs spared) is consistent with differential susceptibility to Complement-mediated attack. These observations collectively imply a plausible involvement of the Complement system within the serum fractions that trigger the swift cell-death response. We therefore conclude that crude alligator serum harbors potent Complement-mediated activity that alligators exploit to rapidly identify and eliminate cancer cells.

### 4.3. From Alligators to Crocodiles and Chickens

Because C5 is a key component of the Complement system [22,23,24], we tested the hypothesis that closely related species respecting their C5 sequence (which bears homology to the alligator protein; comparison was carried out using the ‘Maximum Likelihood’ algorithm, Figure 4A) exhibit alligator-like anti-cancer effects; indeed, the two closely related species, crocodile and chicken (*Gallus gallus*), display alligator-like anti-cancer activity (Figure 4B). Hence, a notable advance here is the extension of the anti-cancer phenomenon beyond alligator to crocodile and chicken sera, guided by phylogenetic relatedness in the Complement C5 sequence. The findings that crocodile and chicken sera kill RPMI 8226 cells rapidly, and that chicken serum fraction (CSa) kills a broad panel of myeloma and leukemia cell lines while sparing human dermal fibroblasts, indicate that the underlying mechanism is not unique to one species. This is consistent with prior work showing strong Complement activity in crocodilian sera [25], supporting the concept that robust innate immune effector mechanisms are a conserved feature in these lineages. Importantly, the chicken results provide a practical and scalable route for future purification and mechanistic dissection without relying on protected crocodilian species.

### 4.4. Mechanistic Dissection in Chicken Serum Implicates the Involvement of Mg^2+^ and Ca^2+^ Ions, Terminal Complement Proteins and IgM in the Anti-Cancer Activity

**Figure 7:**
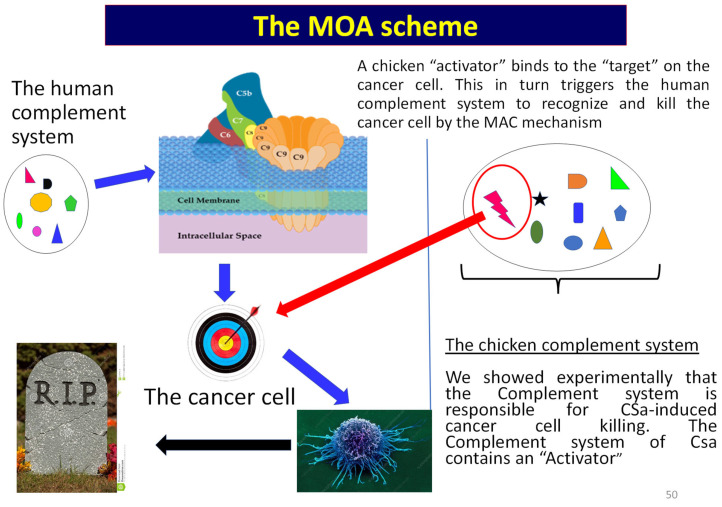
Proposed mechanism of Complement-mediated cancer cell killing. IgM antibodies bind to tumor-associated targets on cancer cells, triggering activation of the classical Complement pathway. This leads to assembly of the membrane attack complex (MAC), resulting in pore formation, membrane disruption, and rapid cell lysis.

Regarding CSa, three mechanistic lines of evidence support Complement dependence: (i) rapid membrane depolarization toward 0 mV; (ii) strong inhibition of killing by EGTA chelation with restoration by Mg^2+^/Ca^2+^, which are required for Complement pathway activity; and (iii) immuno-depletion of terminal Complement proteins (C5–C8) attenuates cytotoxicity. These results strongly favor a model in which Complement activation proceeds to MAC assembly as the proximate executor of tumor cell death (Figure 7). Upstream, IgM appears to participate materially: immuno-depletion of chicken IgM reduced killing by ~82%, and an IgM-enriched chicken fraction can cooperate with human serum to induce activation of the human Complement system, resulting in cell death, consistent with IgM-mediated activation of the classical pathway via C1q binding. This interpretation is well supported by structural and biochemical literature demonstrating how IgM engages C1/C1q to initiate the classical Complement cascade [26,27,28,29]. Accordingly, a plausible unifying mechanism is: (i) chicken (or crocodilian) monomeric IgM (and potentially other “activator” components) binds a cancer-associated target on susceptible cells; (ii) this recruits/activates the Complement (including in heterologous human serum); and (iii) MAC pores drive rapid ionic collapse and lysis.

### 4.5. Study Limitations and Future Directions

Although this study provides experimental evidence supporting the conclusion of a Complement-mediated mechanism underlying cancer cell killing, several limitations are acknowledged:

(1) To further support the ASa and CSa selective killing of cancer cells, additional normal cell types should be tested.
(2) The molecular determinants underlying tumor recognition were not identified. Although our findings provide evidence that IgM antibodies are key initiators of Complement activation, the specific tumor-associated antigens or surface targets triggering this response have not been discovered. Elucidating these targets will be essential for understanding selectivity and for enabling rational therapeutic development.
(3) The following are the limitations with respect to the melanoma and leukemia murine models: (i) The in vivo sample size was limited and did not enable proper statistical analysis. (ii) The exploratory experiments were intended as proof-of-concept preliminary studies, rather than definitive efficacy studies. (iii) Accordingly, these experiments yielded quantitative data.
(4) The in vivo melanoma and lymphoma models only demonstrate proof-of-concept efficacy following intratumoral or intraperitoneal administration, and should be further established once the pure anti-cancer molecule is discovered. This approach does not address the challenges associated with systemic delivery; the pharmacokinetics, biodistribution, and stability of serum-derived components remain unknown, and their behavior in more clinically relevant settings requires further investigation. Finally, potential safety concerns related to Complement activation should also be explored.
(5) While non-malignant human cells were largely spared under the conditions that killed cancer cells, the mechanisms that prevent off-target Complement-mediated damage in vivo should be investigated. Given the potent cytolytic capacity of the Complement system, careful evaluation of toxicity, immunogenicity, and tissue specificity will be critical.
(6) While the enrichment of the Complement component C5 in the active fractions and depletion studies supports the involvement of the Complement system, it is important to note that C5 is an abundant serum protein, and therefore, its identification by proteomic analysis alone does not establish causality but rather should be interpreted in the context of complementary functional evidence. Therefore, the precise contribution of individual Complement components and regulatory factors should be dissected in future studies.
(7) Future experiments may include: recombinant protein reconstitution, Complement inhibition assays and targeted depletion and rescue experiments.
(8) Finally, interspecies differences in immune components may influence translational applicability. While cross-species compatibility was demonstrated in part (e.g., chicken IgM activating human Complement), further work is needed to assess how these findings translate into fully humanized systems.

Importantly, addressing these limitations will be essential to advance this discovery toward clinical application, and to determine whether Complement-based strategies can be safely and effectively harnessed for human cancer therapy.

## 5. Conclusions

This study reveals an ancient, conserved Complement-dependent immune mechanism in alligator, crocodile and chicken sera that selectively kill cancer cells via IgM-triggered MAC formation. The cross-species functionality and absence of toxicity toward healthy mammalian cells highlight this system’s evolutionary sophistication and potential therapeutic relevance. By harnessing this natural immune mechanism, it may be possible to develop bio-inspired, Complement-driven immuno-therapeutics that emulate the precision and potency of these ancient immune strategies, thereby offering a new frontier in cancer immunotherapy. 

## Figures and Tables

**Table 1:** Selected peptides, 10–30 amino acids in length, of relevant chicken Complement proteins used to immunize rabbits for raising polyclonal antibodies.

Protein Name	Epitopes Selected
Complement C5	GGKCSEVIRTKRSDFEEQVLKE, RDDGYRGEDGGPLGRLEACAKYRPSRREP
Complement C6	RTVRRNETRKSYRVPAN, PINGGKPCKGEREEEED
Complement C7	EEDGTDEDQCE, NSGHSYSEKKNEQQSR
Complement C8α	LLVILDPVPVGLT, KEEMRRKDIS
Complement C8β	RRRQCNNPTPQNGGS, RKPYNVESYTPETKGKY
Complement C8γ	SLGELVGRWFL

## Data Availability

All new data are included in the manuscript.

## Supplementary Materials

The following supporting information can be downloaded at: https://www.mdpi.com/article/10.3390/cells15090749/s1. Figure S1. Intratumoral injection of ASa suppresses melanoma growth in mice. (A) Representative histological sections of B16 melanoma tumors from a mouse treated with FCS or ASa. ASa-treated tumors show extensive necrosis and loss of tumor architecture compared to controls. Note, in the FCS-treated mouse, the melanoma cells have large nuclei and a lot of melanin, which is typical to this cell line. In contrast, in the ASa-treated mouse most cancer cells are dead, circled by the red line. See text for details. (B) Quantification of tumor volume over time. ASa treatment reduces tumor growth by ~50% compared to untreated and FCS-treated mice. Figure S2. Intraperitoneal administration of ASa suppresses lymphoma growth in mice. See Materials and Methods for details. The Y-axis shows the percentage of the remaining EL4 cells in the ascites fluid during the experiment. The values were calculated as follows: (EL4 cell count in the ASa-treated mice/EL4 cell count in the FCS-treated mouse) × 100. As shown, 4 days after ASa injection, EL4 cell count was decreased by ~80% compared to the cell count in FCS-injected lymphoma mice.

## Abbreviations

The following abbreviations are used in this manuscript:

AS	Alligator serum
ASa	(NH_4_)_2_SO_4_-precipitated alligator serum
CS	Chicken serum
CSa	(NH_4_)_2_SO_4_-precipitated alligator serum
iPSC-CMs	Induced pluripotent-stem-cell-derived cardiomyocytes
MTT	3-(4,5-dimethylthiazol-2-yl)-2,5-diphenyltetrazolium bromide
MAC	Membrane attack complex

## References

[1] Siddiqui R., Mansoor S., Khan N.A. (2016). Do crocodiles and alligators hold the key to cancer treatment?. BMJ.

[2] Siddiqui R., Jeyamogan S., Ali S.M., Abbas F., Sagathevan K.A., Khan N.A. (2017). Crocodiles and alligators: Antiamoebic and antitumor compounds of crocodiles. Exp. Parasitol..

[3] Khan N.A., Soopramanien M., Siddiqui R. (2019). Crocodiles and alligators: Physicians’ answer to cancer?. Curr. Oncol..

[4] Ghosh S., Lai J.-Y. (2024). An insight into the dual role of MoS2-based nanocarriers in anticancer drug delivery and therapy. Acta Biomater..

[5] Lehrer R.I., Ganz T. (2002). Defensins of vertebrate animals. Curr. Opin. Immunol..

[6] Zasloff M. (2002). Antimicrobial peptides of multicellular organisms. Nature.

[7] Deslouches B., Di Y.P. (2017). Antimicrobial peptides with selective antitumor mechanisms: Prospect for anticancer applications. Oncotarget.

[8] Hoskin D.W., Ramamoorthy A. (2008). Studies on anticancer activities of antimicrobial peptides. Biochim. Biophys. Acta Biomembr..

[9] Merchant M.E., Roche C., Elsey R.M., Prudhomme J. (2003). Antibacterial properties of serum from the American alligator (*Alligator mississippiensis*). Comp. Biochem. Physiol. Part B Biochem. Mol. Biol..

[10] Jeyamogan S., Khan N.A., Sagathevan K., Siddiqui R. (2020). *Crocodylus porosus*: A potential source of anticancer molecules. BMJ Open Sci..

[11] Yehezkel S., Rebibo-Sabbah A., Segev Y., Tzukerman M., Shaked R., Huber I., Gepstein L., Skorecki K., Selig S. (2011). Reprogramming of telomeric regions during the generation of human induced pluripotent stem cells and subsequent differentiation into fibroblast-like derivatives. Epigenetics.

[12] Lian X., Zhang J., Azarin S.M., Zhu K., Hazeltine L.B., Bao X., Hsiao C., Kamp T.J., Palecek S.P. (2013). Directed cardiomyocyte differentiation from human pluripotent stem cells by modulating Wnt/β-catenin signaling under fully defined conditions. Nat. Protoc..

[13] Neeman-Egozi S., Livneh I., Dolgopyat I., Nussinovitch U., Milman H., Cohen N., Eisen B., Ciechanover A., Binah O. (2024). Stress-induced proteasome sub-cellular translocation in cardiomyocytes causes altered intracellular calcium handling and arrhythmias. Int. J. Mol. Sci..

[14] Shiver J.W., Dankert J.R., Esser A.F. (1991). Formation of ion-conducting channels by the membrane attack complex proteins of complement. Biophys. J..

[15] Jones J., Morgan B.P. (1990). Transience of membrane channels induced by perforin and by the membrane attack complex. Biochem. Soc. Trans..

[16] Zalman L.S., Müller-Eberhard H.J. (1990). Comparison of channels formed by poly C9, C5b-8 and the membrane attack complex of complement. Mol. Immunol..

[17] Binah O., Liu C.-C., Young J.D.-E., Berke G. (1997). Channel Formation and [Ca^2+^]_i_ accumulation induced by perforin N-terminus peptides: Comparison with purified perforin and whole lytic granules. Biochem. Biophys. Res. Commun..

[18] Aleshin A.E., DiScipio R.G., Stec B., Liddington R.C. (2012). Crystal structure of C5b-6 suggests structural basis for priming assembly of the membrane attack complex. J. Biol. Chem..

[19] Dudkina N.V., Spicer B.A., Reboul C.F., Conroy P.J., Lukoyanova N., Elmlund H., Law R.H.P., Ekkel S.M., Kondos S.C., Goode R.J.A. (2016). Structure of the poly-C9 component of the complement membrane attack complex. Nat. Commun..

[20] Serna M., Giles J.L., Morgan B.P., Bubeck D. (2016). Structural basis of complement membrane attack complex formation. Nat. Commun..

[21] Menny A., Serna M., Boyd C.M., Gardner S., Joseph A.P., Morgan B.P., Topf M., Brooks N.J., Bubeck D. (2018). CryoEM reveals how the complement membrane attack complex ruptures lipid bilayers. Nat. Commun..

[22] Merchant M.E., Verret B., Elsey R.M. (2005). Role of divalent metal ions in serum complement activity of the American alligator (*Alligator mississippiensis*). Comp. Biochem. Physiol. Part B Biochem. Mol. Biol..

[23] Hadders M.A., Bubeck D., Roversi P., Hakobyan S., Forneris F., Morgan B.P., Pangburn M.K., Llorca O., Lea S.M., Gros P. (2012). Assembly and regulation of the membrane attack complex based on structures of C5b6 and sC5b9. Cell Rep..

[24] Ricklin D., Hajishengallis G., Yang K., Lambris J.D. (2010). Complement: A key system for immune surveillance and homeostasis. Nat. Immunol..

[25] Merchant M., Britton A. (2006). Characterization of serum complement activity of saltwater (*Crocodylus porosus*) and freshwater (*Crocodylus johnstoni*) crocodiles. Comp. Biochem. Physiol. Part A Mol. Integr. Physiol..

[26] Gaboriaud C., Thielens N.M., AGregory L., Rossi V., Fontecilla-Camps J.C., Arlaud G.J. (2004). Structure and activation of the C1 complex of complement: Unraveling the puzzle. Trends Immunol..

[27] Merle N.S., Church S.E., Fremeaux-Bacchi V., Roumenina L.T. (2015). Complement System Part I—Molecular Mechanisms of Activation and Regulation. Front. Immunol..

[28] Thielens N.M., Tedesco F., Bohlson S.S., Gaboriaud C., Tenner A.J. (2017). C1q: A fresh look upon an old molecule. Mol. Immunol..

[29] John M.M., Barthel A., Hawlin V., Wozniak-Knopp G., Kunert R. (2026). Engineering C1q single-chain globular head variants for enhanced IgM binding. BMC Biol..

