# Supplementary material for: Sera from Phylogenetically Related Alligators, Crocodiles and Domestic Chickens Exhibit Comparable Anti-Cancer Activity
Source: Cells. 2026 Apr 22;15(9):749. doi: 10.3390/cells15090749 (PMC13162711; doi:10.3390/cells15090749)
Supplement: Supplementary file 1 [file cells-15-00749-s001.zip › cells-4241330-supplementary.pdf]

Supplementary Material

Sera from Phylogenetically-Related Alligators, Crocodiles and Domestic Chickens Exhibit a Comparable Anti-Cancer Activity

Ofer Binah [1,†,*], Gil Shalev [2,†], Gila Maor [1], Irina Reiter [1], Inbal Ziv [2] and Aaron Ciechanover [2,*]

[1]Department of Physiology, Biophysics and Systems Biology, Rappaport Faculty of Medicine, Technion-Israel Institute of Technology, Haifa 3190601, Israel.
[2]The Rappaport-Technion Integrated Cancer Center (R-TICC) and The Rappaport Faculty of Medicine and Research Institute, Technion-Israel Institute of Technology, Haifa 3109601, Israel.
*Correspondence: AC, aaroncie@technion.ac.il; OB, binah@technion.ac.il.
[†]These authors are equal contributors.

1. Materials and Methods

1.1. The Melanoma Model in C57/bl Mice

Melanoma tumor was induced by intracutaneous injection of murine B16 (melanoma-derived) cells (donated by the late Prof. Yoram Palti, The Technion - Israel institute of Technology) into the back skin of C57/bl mice (Fig. S1A). Three days after B16 injection when the tumor became palpable, mice were separated into 3 groups: ASa-treated-, FCS-treated and non-treated mice. In the two treated groups, ASa and FCS were injected (100 μl) intratumorally throughout the 8-day experiment, at 48-hour intervals (4 injections). Tumor dimensions were measured on days 1, 2, 3, 6, 7 and 8 with a digital caliper, and the volumes were calculated assuming tumors are roughly cuboidal. On the 8th day, tumors were excised, histologically processed and stained with Hematoxylin-Eosin.

1.2. The Lymphoma Model in C57/bl Mice

Murine syngeneic peritoneal lymphoma was generated by injecting $0.5x10^6$ EL4 cells intraperitoneally (i.p.) into inbred strain C57Bl/6 mice. Three days after the EL4 injection (Fig. S2), when the EL4 cell count was ~5-$8x10^6$, FCS or ASa (45% $(NH_4)_2SO_4$ precipitation of alligator serum) were injected i.p. at a dose of 100 μl/10^6 EL4 cells. Following the first FCS or ASa injection, both were injected at 24, 48 and 72 hrs (one ASa and one FCS-injected mouse). At the time points 0, 24, 48, 72 and 96 hrs, one mouse was sacrificed from each group, ascites fluid was collected and EL4 count measured.

2. Results

2.1. ASa causes anti-tumor effects in in vivo models of murine Melanoma and Lymphoma

In the melanoma model, ASa-treated tumors show extensive necrosis and loss of tumor architecture compared to controls (Fig. S1A). In the FCS-treated mouse, the melanoma cells have large nuclei and a lot of melanin, which is typical to this in vivo melanoma model. In contrast, in ASa-treated mice, most cancer cells are dead, circled by the red line. As shown in Fig. S1B, on day 8 ASa decreased the tumor volume by ~50% compared to control and FCS-injected tumors. In the lymphoma model (Fig. S2), 4 days after ASa injection, EL4 cell count was decreased by ~80% compared to the cell count in the FCS-injected lymphoma mouse.

Figure S1

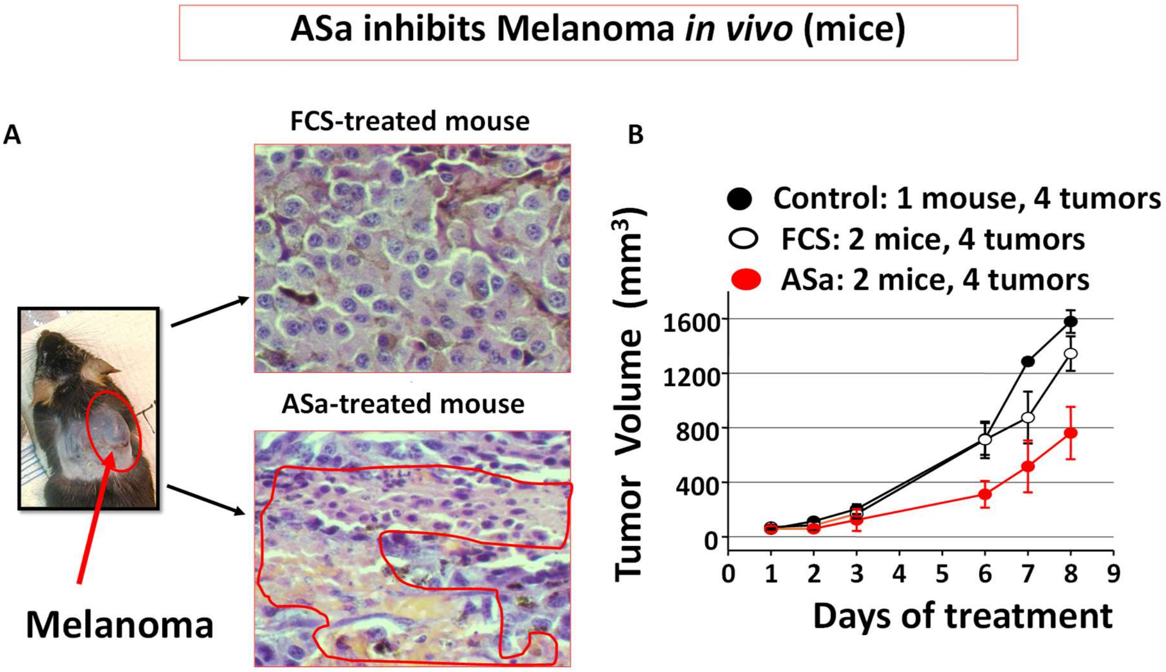

Figure S1. Intratumoral injection of ASa suppresses melanoma growth in mice. (A) Representative histological sections of B16 melanoma tumors from a mouse treated with FCS or ASa. ASa-treated tumors show extensive necrosis and loss of tumor architecture compared to controls. Note, in the FCS-treated mouse, the melanoma cells have large nuclei and a lot of melanin, which is typical to this cell line. In contrast, in the ASa-treated mouse most cancer cells are dead, circled by the red line. See text for details. **(B)** Quantification of tumor volume over time. ASa treatment reduces tumor growth by ~50% compared to untreated and FCS-treated mice.

Figure S2

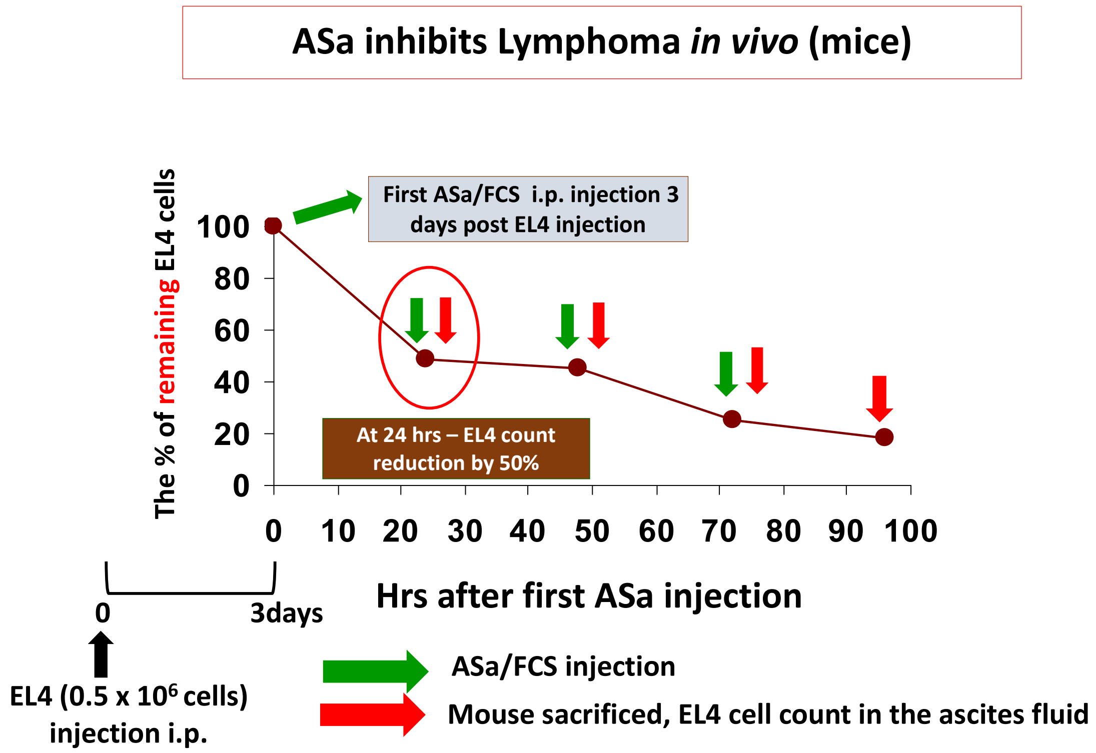

Figure S2. Intraperitoneal administration of ASa suppresses lymphoma growth in mice. See Materials and Methods for details. The Y-axis shows the percentage of the remaining EL4 cells in the ascites fluid during the experiment. The values were calculated as follows: (EL4 cell count in the ASa-treated mouse/EL4 cell count in the FCS-treated mouse) x 100. As shown, 4 days after ASa injection, EL4 cell count was decreased by ~80% compared to the cell count in FCS-injected lymphoma mouse.
